# Percutaneous Left Atrial Appendage Occlusion: An Emerging Option in Patients with Atrial Fibrillation at High Risk of Bleeding

**DOI:** 10.3390/medicina57050444

**Published:** 2021-05-03

**Authors:** Giovanni Cimmino, Francesco S. Loffredo, Emanuele Gallinoro, Dario Prozzo, Dario Fabiani, Luigi Cante, Gemma Salerno, Maurizio Cappelli Bigazzi, Paolo Golino

**Affiliations:** 1 Department of Translational Medical Sciences, University of Campania Luigi Vanvitelli, 80131 Naples, Italy; giovanni.cimmino@unicampania.it (G.C.); e.gallinoro@gmail.it (E.G.); dario.prozzo@gmail.com (D.P.); dario.fabiani94@gmail.com (D.F.); luigicante3@gmail.com (L.C.); paolo.golino@unicampania.it (P.G.); 2 Vanvitelli Cardiology Unit, Monaldi Hospital, 80131 Naples, Italy; gemma.salerno@hotmail.it (G.S.); mcappellibigazzi@gmail.com (M.C.B.); 3 Molecular Cardiology, International Centre for Genetic Engineering and Biotechnology, 34149 Trieste, Italy; 4 Cardiovascular Center Aalst, OLV Clinic, 9300 Aalst, Belgium

**Keywords:** atrial fibrillation, cardioembolism, stroke prevention, bleeding risk, left atrial appendage occlusion

## Abstract

Atrial fibrillation (AF) is a common cardiac arrhythmia with an estimated prevalence of 1% in the general population. It is associated with an increased risk of ischemic stroke, silent cerebral ischemia, and cognitive impairment. Due to the blood flow stasis and morphology, thrombus formation occurs mainly in the left atrial appendage (LAA), particularly in the setting of nonvalvular AF (NVAF). Previous studies have shown that >90% of emboli related to NVAF originate from the LAA, thus prevention of systemic cardioembolism is indicated. According to the current guidelines, anticoagulant therapy with direct oral anticoagulants (DOACs) or vitamin K antagonists (VKAs), represents the standard of care in AF patients, in order to prevent ischemic stroke and peripheral embolization. Although these drugs are widely used and DOACs have shown, compared to VKAs, non-inferiority for stroke prevention with significantly fewer bleeding complications, some issues remain a matter of debate, including contraindications, side effects, and adherence. An increasing number of patients, indeed, because of high bleeding risk or after experiencing life-threatening bleedings, must take anticoagulants with extreme caution if not contraindicated. While surgical closure or exclusion of LAA has been historically used in patients with AF with contradictory results, in the recent years, a novel procedure has emerged to prevent the cardioembolic stroke in these patients: The percutaneous left atrial appendage occlusion (LAAO). Different devices have been developed in recent years, though not all of them are approved in Europe and some are still under clinical investigation. Currently available devices have shown a significant decrease in bleeding risk while maintaining efficacy in preventing thromboembolism. The procedure can be performed percutaneously through the femoral vein access, under general anesthesia. A transseptal puncture is required to access left atrium and is guided by transesophageal echocardiography (TEE). Evidence from the current literature indicates that percutaneous LAAO represents a safe alternative for those patients with contraindications for long-term oral anticoagulation. This review summarizes scientific evidences regarding LAAO for stroke prevention including clinical indications and an adequate patient selection.

## 1. Introduction

AF is the most frequent cardiac arrhythmia in elderly, affecting up to the 15% of patients older than 80 years. It is associated with an annual risk of major stroke greater than 5% [1]. Compared to the general population, this risk increases of five-to seven-fold and it may lead to silent cerebral ischemia and cognitive dysfunction [2]. Anticoagulation is the gold standard strategy for stroke prevention: DOACs are recommended as a first-line therapy in NVAF patients, while VKAs are used in AF patients with mechanical heart valves or moderate-to-severe mitral stenosis [1]. However, the lifelong dependence from anticoagulation in AF patients is inevitably associated with an increased risk for bleeding complications as well as cardioembolic events in case of inadequate therapy and, finally, to significant lifestyle modifications (e.g., talking to the healthcare provider before taking medications, herbals, or supplements, performing periodic blood tests, and avoiding major diet changes when taking VKAs) [3]. Autopsy and transesophageal echocardiographic (TEE) studies have shown that, most of the thrombi AF-related, are localized in LAA [4], indicating LAA as a target for a stroke prevention strategy [5]. As common practice, many AF patients undergoing cardiac surgery (mitral valve surgery or coronary bypass [6]) have undergone to surgical ligation or excision of LAA [7]. However, the high rate of incomplete surgical LAAO together with controversial data available from literature, made it difficult to establish a clear connection between LAAO and prevention of cardioembolic stroke [8]. Results from randomized clinical trials (RCTs) have clearly indicated that anticoagulation is the first-line treatment for stroke prevention in AF patients [1]. However, in the last two decades, several papers discussing the role of percutaneous LAAO in stroke prevention have been published [9], increasing the interest in this procedure as an alternative to anticoagulant therapy in NVAF patients [10]. Conversely, in AF patients with mechanical heart valves or moderate-to-severe mitral stenosis, VKAs therapy represents the gold standard since thrombus formation occurs also outside the LAA and percutaneous LAAO may not provide an adequate protection from embolic stroke. The growing experience accumulated in the last five years, supported by recent clinical trials reporting the safety and the non-inferiority of percutaneous LAAO in the prevention of cardioembolic stroke compared to long-term anticoagulation, indicate that this therapeutic approach is becoming an important alternative in the management of the cardioembolic risk in AF patients. In the present narrative review, we will discuss the emerging role of percutaneous LAAO in stroke prevention focusing on technical aspects of the procedure and current indications.

## 2. Pathophysiology of Thrombus Formation in LAA during AF

LAA is a tubular structure trabeculated, that has a great variability in sizes and shapes [4]. Anatomically, it is divided into three regions: ostium, neck, and a lobe [11]. LAA is an embryonic remnant that contributes, through its great adaption to pressure and volume overload, to left diastolic ventricular filling thanks to its reservoir function [12]. LAA has also an important endocrine function, participating to the production and secretion of atrial natriuretic peptide (ANP) and brain natriuretic peptide (BNP) [13]. Of note, some reports indicate that LAAO has been associated to a reduction of serum levels of ANP and BNP [14,15]. LAA of patients with AF is subjected to several changes, including dilatation, stretching, and a reduction in pectinate muscles volume [16].

These changes may explain in part why the source of an embolic events in AF patients is often a thrombus localized in the LAA [10,17]: it is probably the result of the interaction between three components: blood stasis, changes in the inner layer of the atrium and hemostasis abnormalities as for Virchow triad [18,19]. The main mechanisms of thrombogenesis are the following:

(1) Atrial remodeling in AF patients promotes stasis and increases the risk of thromboembolic events [20]. Spontaneous echocardiographic contrast, seen during echocardiographic imaging, is an independent predictor for stroke in AF patients [21].
(2) Alterations of the inner layer of the LAA in AF patients may promote thrombogenesis due to structural abnormalities that may occur during the implantation procedure [18,19].
(3) Many molecular pathways (inflammation, growth factors, nitric oxide, and the renin-angiotensin-aldosterone system) can contribute to promoting a prothrombotic state in AF patients [22,23].

## 3. Percutaneous LAAO Procedure

Percutaneous LAAO can be performed through an endocardial approach, less often using a hybrid epicardial/endocardial approach, under TEE guidance with general anesthesia, that ensures complete immobility of patients to reduce the risk of mechanical complications [24]. The right femoral vein is the preferential access site although the use of left femoral vein has been reported in patients with unavailable right access (e.g., previous vascular surgery, arteriovenous fistula, etc.) [25]. The transseptal puncture is a crucial phase of percutaneous LAAO procedure and it is preferably performed in the postero-inferior area of interatrial septum because LAA, in most cases, is oriented anterolaterally and superiorly. Nevertheless, it is possible to access into LAA using a pre-existing patent foramen ovale (PFO) or an atrial septal defect (ASD), but only in case of a favorable LAA orientation (lateral or posterior) [26].

Optimal pre-procedural LAAO imaging has several advantages: to rule out LAA thrombus, to detail LAA anatomy, to assess surrounding structures (e.g., interatrial septum or pulmonary veins), to measure LAA dimensions in order to select type and size of the device and to determine the location of transseptal puncture. TEE and cardiac computed tomography angiography (CCTA) currently represent the main imaging for LAAO pre-procedural planning. Although TEE has been considered traditionally the gold-standard pre-procedural imaging for LAAO, CCTA has showed the following advantages: a superior spatial resolution, a noninvasive data acquisition and a detailed three-dimensional (3D) characterization of the LAA anatomy to predict the correct size of the closing device [27]. Interestingly, some studies have demonstrated the superiority of CT-based 3D imaging compared to TEE particularly in case of challenging LAA anatomies [28,29]. The 3D acquisition technology offers the possibility of using virtual reality to reconstruct LAA geometry and to simulate virtual implantation of percutaneous devices [30].

**Figure 1:**
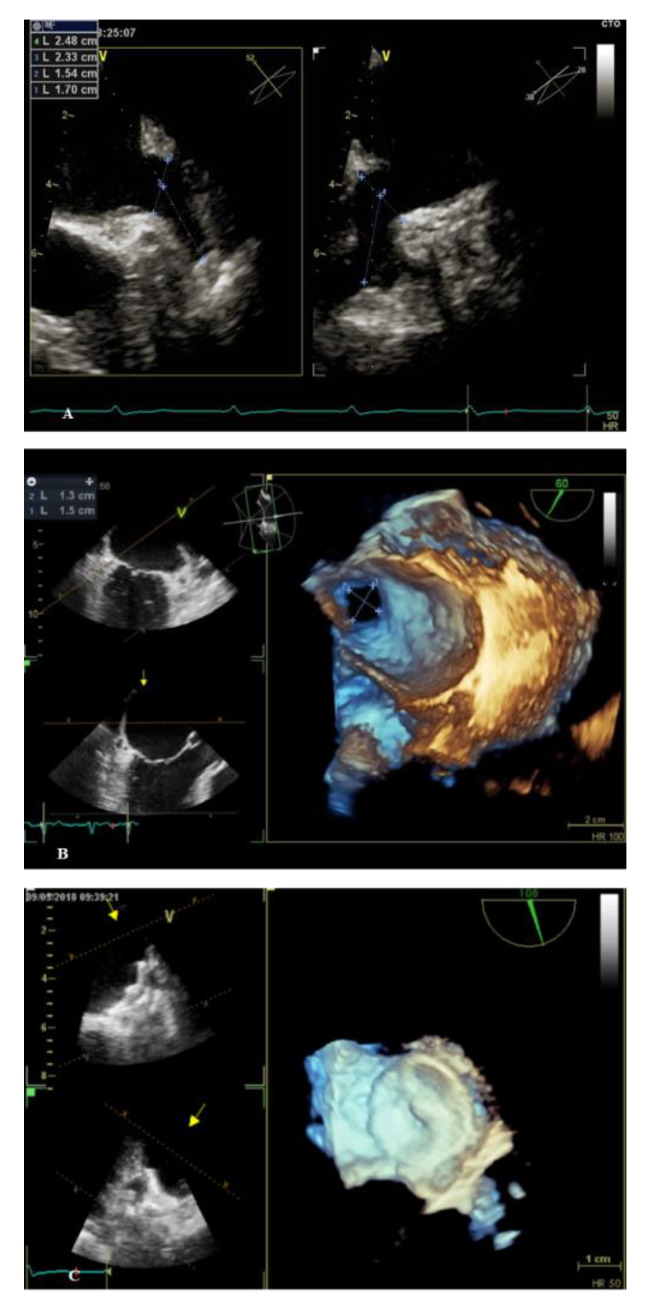
Examples of LAAO evaluation by 2D and 3D transesophageal echocardiography performed in our unit. (**A**) 2D-TEE evaluation of LAA; (**B**) 3D-TEE assessment of LAA; (**C**) 3D-TEE evaluation of an implanted LAA device.

Before or upon transseptal puncture, heparin is administered intravenously to achieve a target of active clotting time (ACT) >250 s [17]. TEE allows to exclude presence of thrombotic formations in LAA, to guide the transseptal puncture and, finally, to verify the correct position of the device and the presence of peri-device leaks [31] as shown in Figure 1. Several meta-analysis have assessed successes and complications of this procedure. Two recent meta-analysis [9,32], including a total of 3585 and 12,415 patients, have reported a failure rate of 2%, periprocedural stroke and cardiac tamponade in 1% and 2% of patients, respectively, mortality rate of 0.28% during and after this procedure, a periprocedural stroke risk of 0.31%, with an incidence of procedural-related severe bleeding and pericardial effusion of 1.71% and 3.25%. After discharge, post-procedural imaging using TEE or CCTA should be recommended at 6–24 weeks after device implantation to evaluate the presence of device-related thrombus or peri-device leaks [26].

Device-related thrombus is an independent predictor factor of stroke/TIA and is associated with a higher risk of stroke and systemic embolism [33]. The incidence of device-related thrombus has been reported between 2% and 4% in several studies. In the PROTECT-AF trial [34], device-related thrombus was observed in 3.4% of patients after one year of follow-up. In the ACP registry [35] was reported a prevalence of 3.2% of patients after a median follow-up of 134 days, while the EWOLUTION registry [36] has showed a rate of 2.6% at three months. Pharmacological therapy after device implant (with OAC or antiplatelet drugs) contributes to reduce the risk of device-related thrombus [33]. Since cases of very late device-related thrombus (after >12 months) have been reported, imaging may be repeated 12 months after device implantation and should be treated with proper anticoagulation until resolution of thrombus [26].

Peri-device leaks contribute to create a connection with LAA allowing thrombi to enter into the systemic circulation. Peri-device leaks < 5 mm are considered irrelevant and may close spontaneously. Leaks > 5 mm, based on PROTECTF-AF [34] data, require OAC or a second occlusion procedure, although a clear benefit using this strategy has not been proved [26]. Incidence of peri-device leaks is variable in different studies: in PROTECT-AF [34] was observed in 32% of patients after one year, while in EWOLUTION [36] registry the incidence of leaks >5 mm occurred only in 1% of cases at three months. Peri-device leaks can be studied both by TEE and CCTA, with the latter that appears a valid alternative. In this direction, a number of trials have compared these two imaging techniques. Based on these studies, CCTA has shown a higher diagnostic accuracy than TEE to detect peri-device leaks [37]. Therefore, CCTA appears as a feasible alternative to TEE for follow-up post-implantation, to evaluate not only the presence and severity of peri-device leaks, but also other complications, such as device thrombus, embolization, and pericardial effusion [38]. Nevertheless, the presence of peri-device leaks has not demonstrated to correlate to clinical events [39] and its assessment is not reported in the most recent recommendations [26].

## 4. Devices Characteristics

Several devices have been tested in the last decade and some of those are currently available for clinical use (Table 1) [40]. Percutaneous LAAO through endocardial approach is the most commonly used method, followed by a period of dual antiplatelet therapy (DAPT) of variable duration [41], in order to prevent device-related thrombus.

The first percutaneous LAAO was performed in 2001 using the PLAATO device (Appriva Medical, Sunnyvale, CA, USA), that was formed by a self-expanding metal cage of different sizes (15–32 mm). Despite of positive initial results [42], this device has no longer been available since 2006, due to a high risk of procedure-related complications and residual shunts [43].

The Watchman™ (Boston Scientific, Natick, MA, USA) and the Amplatzer Amulet™ (Abbott Laboratories, Chicago, IL, USA) are two devices with the largest evidences of safety and effectiveness [44]. In addition to these options, percutaneous LAAO can be performed with an endo-epicardial approach, using the LARIAT^®^ device (SentreHeart, Inc., Redwood City, CA, USA) with good results in terms of efficacy and safety [45]. Two more devices for epicardial LAAO are still under evaluation: Sierra Ligation System (Aegis Medical, Los Angeles, CA, USA) and TigerPaw Pro (Laax, Livermore, CA, USA) (TPP) [46].

Watchman™. This is the first percutaneous device approved by the US Food and Drug Administration (FDA) to perform LAAO in NVAF patients through an endocardial approach [36]. It consists of a self-expanding prothesis with a nitinol cage coated by a permeable polyester structure and anchored with fixation barbs on the atrial surface. Various sizes are available (21–33 mm).

Amplatzer Cardiac Plug (AGA, St. Jude Medical, Minneapolis, MN, USA) (ACP). The ACP is a self-expanding prothesis formed by a nitinol mesh. It is constituted by a distal lobe of various sizes (16–30 mm) with fixation barbs and a proximal disk that is positioned to cover the LAA mouth. A short central waist connects the proximal and the distal region of this device [35,47,48].

Amplatzer Amulet^®^ (Abbott Laboratories, Chicago, IL, USA). This is a second-generation device of the Amplatzer. Compared to ACP, it presents a higher number of sizes (up to 34 mm) and stabilizing wires, with comparable safety and efficacy [47,48,49]. A number of studies evaluating ACP and Amulet have shown a successful rate of device implantation in the majority of patients, with a low rate of major periprocedural complications (death, stroke/TIA, device embolization, perforation/tamponade/effusion, and major bleeding) [50,51,52].

Lariat^®^ (SentreHEART, Redwood City, CA, USA) consists of LAA ligation using an endo-epicardial approach where surgical sutures are introduced in the pericardium and ligated percutaneously around the LAA ostium. This approach may be considered when LAA is too large for endocardial occlusion devices. Antiplatelet therapy is not necessary in this case because of the absence of an endocardial device [45,53].

An overview of the devices currently available is provided in Table 1.

## 5. Randomized Clinical Trials Evaluating Safety and Efficacy of Percutaneous LAAO

PROTECT AF. It was the first RCT evaluating percutaneous LAAO efficacy and safety [34]. In this study, 707 patients with NVAF and CHA2DS2-VASc score >1 were assigned to receive percutaneous LAAO (*n* = 463) or medical therapy with warfarin (OAC, *n* = 244). Patients with contraindications to warfarin were excluded. LAAO was carried out with the Watchman device. In this study the primary efficacy endpoint consisted of a composite of stroke, systemic embolism (SE) or cardiovascular/unexplained death with an events rate in LAAO group (2.3 per 100 patient-years) lower than OAC group (3.8 per 100 patient-years). Although ischemic stroke events were numerically more frequent in the LAAO group compared to OAC, this difference was not statistically relevant. The primary safety endpoint was a composite of major bleeding and procedure-related complications. The rate of hemorrhagic stroke was lower in the Watchman arm (0.6%) compared to warfarin (1.1%): this result differed from others RTCs using a warfarin group as stroke prevention strategy in AF patients (0.38% in RELY [54], 0.7% in ROCKET-AF [55], 0.47% in ARISTOTLE [56] and 0.47% in ENGAGE AF-TIMI 48 [57]) as shown in Table 2.The device was successfully implanted in 90.9% of cases with low incidence of complications: cardiac tamponade (5%), major bleeding (3%), pericardial effusions (1.7%), periprocedural stroke (1%), and device embolization (0.6%).

PREVAIL. The second randomized study to investigate the efficacy and the safety of the Watchman closure system, compared to OAC therapy, was the PREVAIL (Prospective Randomized Evaluation of the Watchman Left Atrial Appendage Closure Device in Patients with Atrial Fibrillation versus Long-Term Warfarin Therapy) [58]. This trial enrolled 407 NVAF patients who had a higher thromboembolic risk than patients enrolled in PROTECT-AF [34]: CHADS2 score ≥2 or ≥1 with an additional risk factor: female sex, age ≥75 years, baseline ejection fraction between 30% and 35%, age 65–74 years and either diabetes or coronary artery disease and age >65 years with congestive heart failure. Patients were randomized into two groups to receive percutaneous LAAO (*n* = 269) or OAC therapy (*n* = 138). The first coprimary efficacy endpoint, who was a composite of ischemic or hemorrhagic stroke, SE, and cardiovascular/unexplained death, did not achieve non-inferiority criteria: the rate of events in warfarin group was similar to watchman group (RR: 1.07; 95% CrI: 0.57–1.89). This result may be explained considering a lower rate of ischemic stroke (0.71 per 100 patient years) in the warfarin group of the PREVAIL when compared to others more recent RCTs (1.69% in RELY [54], 2.2% in ROCKET-AF [55], 1.6% in ARISTOTLE [56] and 1.50% in ENGAGE AF-TIMI 48 [57]) as shown in Table 2. These differences may be related to different percentages of time in therapeutic range observed in these studies (68% in PREVAIL in comparison to 64% in RELY [54], 55% in ROCKET-AF [55], and 62% in ARISTOTLE [56]). The small sample size of this trial could have contributed to the final result. The second coprimary efficacy endpoint, a composite of ischemic stroke or SE, excluding the first seven days post-device implantation, achieved non-inferiority criteria. Early periprocedural events, occurring within seven days from device implantation, were excluded to better evaluate the efficacy of the device to reduce late ischemic stroke and SE events. The primary safety endpoint was evaluated in the device group and was a composite of all-cause death, ischemic stroke, SE or procedure-related events requesting surgery occurred within seven days from the procedure or during the index hospitalization. This endpoint achieved non-inferiority with a rate of events only in 2.2% of patients. The rate of complications decreased from PROTECT-AF [34] (8.7%) to PREVAIL [58] (4.2%) as shown in Table 3, possibly because of increased operator’s experience.

An overview of ARISTOTLE, RELY, ENGAGE-AF TIMI, and ROCKET-AF trials are shown in Table 2.

The five-year outcome data of PROTECT AF and PREVAIL were combined in a meta-analysis [59]. The primary endpoint (a composite of stroke, SE and cardiac death) achieved non inferiority criteria compared to warfarin. In addition, this meta-analysis showed a lower rate of hemorrhagic stroke (HR: 0.20), stroke with severe disability (HR: 0.45), and cardiovascular/unexplained mortality (HR: 0.59) in the Watchman arm. However, a limit of PROTECT AF [34] and PREVAIL [58] is represented by the small number of patients with absolute contraindication to OAC. Furthermore, the control group did not include patients taking DOACs, that represents the standard of care in accordance with the latest ESC guidelines [1].

An overview of PROTECT-AF and PREVAIL trials are shown in Table 3.

PRAGUE-17. Compared to warfarin, DOACs are easier to use and safer with a lower hemorrhagic risk leading to a reduction in mortality [60,61,62]. RCTs evaluating efficacy and safety of percutaneous LAAO started before the widespread use of DOACs over warfarin in AF patients [63,64,65].

Left Atrial Appendage Closure vs. Novel Anticoagulation Agents in Atrial Fibrillation (PRAGUE-17) [66] was the first multicenter, randomized, non-inferiority trial who compared percutaneous LAAO versus DOACs therapy. Patients were assigned in a 1:1 ratio to LAAO group (*n* = 201) and DOACs group (*n* = 201). Different devices have been used in the LAAO group (Amulet, Watchman, and Watchman-FLX in 61.3%, 35.9%, and 2.8% of patients, respectively). In the DOAC group, apixaban was the most used DOACs (95.5% of patients). Inclusion criteria were evidence of NVAF and one of the following: history of bleeding requiring intervention or hospitalization even without OAC treatment, history of a cardioembolic event during OAC treatment, CHA2DS2-VASc ≥3 and HAS-BLED >2. The selected cohort was at high risk of stroke with a mean CHA2DS2-VASc 4.7 +/− 1.5 and at high bleeding risk with a mean HAS-BLED of 3.1 +/− 0.9. The primary endpoint, that was a composite of stroke (any type) or TIA, SE, cardiovascular death, major or non-major clinically-relevant bleeding or procedural/device-related complications, achieved non-inferiority criteria (10.99 per 100 patient/years in the LAAO group vs. 13.42 per 100 patient/years in the DOACs group) as shown in Table 3. On the other hand, there were not statistically significant differences between the two groups for the single components of the primary endpoint: stroke (any type)/TIA (2.6 per 100 patient/years in LAAO group vs. 2.57 per 100 patient/years in the DOACs group), cardiovascular mortality (3.18% vs. 4.28%). The frequency of major/non-major bleeding event was numerically superior in DOAC group compared to Watchman (7.42 vs. 5.5%). Procedural/device-related complications occurred in 4.5% of patients. However, for this trial it should be considered that (1) both Watchman and Amulet were used as closure devices for LAAO. The differences between these (e.g., location of occlusion, residual leak rate, embolization risk, sizes) and the currently absence of a RCTs comparing these two devices, could represent a limitation in data analysis or, on the other hand, could better represent the “real-world” clinical practice vision; (2) because of the small sample size, PRAGUE-17 is underpowered to evaluate the relative differences in the single components of the primary endpoint.

Interestingly, the development or exacerbation of heart failure was not consistently assessed in any of the aforementioned trials regarding percutaneous LAAO and is not planned to be registered as an endpoint in ongoing studies.

## 6. Advanced Data Analysis Exploring the Available Data on LAAO

A meta-analysis by LI et al. [67] compared efficacy and safety of percutaneous LAAO and DOACs, including six RCTs (ARISTOTELE [56], ENGAGE AF-TIMI 48 [57], RE-LY [54], ROCKET-AF [55], PROTECT AF [34], and PREVAIL AF [58]) and 27 observational studies that have been included due to the small number of RCTs addressing this matter. This meta-analysis showed a lower efficacy of LAAO compared to DOACs in stroke prevention at over 1 year of follow-up (OR = 0.86) and a lower rate of major bleeding during follow up in LAAO patients. Instead, a meta-proportional analysis based on observational studies showed that both thromboembolic and major hemorrhagic events were reduced in the LAAO group compared to DOACs group.

A network meta-analysis by Ontario et al. [68] included two RCTs comparing LAAO and warfarin (PROTECTAF [34] and PREVAIL [58]) and five studies comparing DOACs and warfarin (to make an indirect comparison between LAAO and DOACs). Results showed a reduction in stroke risk (OR 0.85; CrI: 0.63–1.05) and all-cause mortality (OR 0.71; CrI: 0.49–1.22) between percutaneous LAAO and DOACs groups. In addition, LAAO was superior to DOACs in preventing hemorrhagic stroke (OR 0.45; CrI: 0.29–0.79).

Another meta-analysis [69] including 16 studies, was performed comparing 1759 LAAO patients versus a fictitious control group from the ATRIA study, the Danish national patient registry, and the results of three studies comparing DOACs with warfarin. The risk of stroke was reduced in percutaneous LAAO group in comparison with the others considered groups: no therapy or aspirin groups (RR 0.34; CI: 0.25–0.46) and warfarin group (RR 0.65; CI: 0.46–0.91). Percutaneous LAAO had a higher risk of stroke with a relative risk of 1.69 and 1.59 when compared to DOACs like dabigatran and apixaban respectively. Device deployment was unsuccessful in 6.1% of patients and overall complications rate was 7.1%. There were no differences in term of efficacy and safety between various types of devices used.

A network meta-analysis including 19 RCTs by Sahay et al. [70] showed a greater efficacy of LAAO versus placebo (stroke/SE: HR 0.24; CI: 0.11–0.52), versus antiplatelet therapy (stroke/SE: HR 0.44; CI: 0.23–0.86) and similar efficacy when compared to DOACs (stroke/SE: HR 1.01; CI: 0.53–1.92). The rate of major bleeding events with LAAO was lower than antiplatelet therapy (HR 0.75; CI: 0.30–1.88) and DOACs (HR 0.80; CI: 0.33–1.94).

## 7. Ongoing Studies Looking to a Close Future

Many RCTs are ongoing to evaluate risks and benefits of percutaneous LAAO compared to DOACs therapy [71].

The ASAP-TOO is a RCT that has enrolled 888 patients who have been randomized into two groups: percutaneous LAAO with Watchman device versus single or dual antiplatelet therapy. All the patients in this study were considered ineligible to an anticoagulation therapy. The primary efficacy endpoint is a composite of ischemic stroke or SE during a follow-up of five years. The primary safety endpoint is a composite of death of all-cause, ischemic stroke, SE, early device or procedure/related events requiring surgery.

The STROKECLOSE is a randomized clinical trial designed to recruit 750 patients with previous intracerebral hemorrhage, divided into two groups: percutaneous LAAO with Amplatzer Amulet or medical therapy. The primary efficacy endpoint is one of the following occurrences: Stroke (ischemic or hemorrhagic), SE, life-threatening or major bleeding, and all-cause mortality.

The CLOSURE-AF (Left Atrial Appendage CLOSURE in Patients with Atrial Fibrillation Compared to Medical Therapy) is a randomized trial that has enrolled 1512 patients with NVAF divided according to the treatment strategy: percutaneous LAAO versus the best medical therapy (including DOACs, antiplatelet, or no therapy). This study has included AF patients at high risk of stroke and at high bleeding risk under OAC or with absolute contraindication to OAC.

The OPTION trial is a prospective, randomized, multi-center clinical trial designed to enroll 1600 patients who have been subjected to AF ablation procedure, divided into two groups: Percutaneous LAAO with WATCHMAN FLX or medical therapy using DOACs. The primary efficacy endpoint is a composite of stroke, SE, and all-cause mortality. The primary safety endpoint is the occurrence of non-procedural bleeding (major bleeding and clinically-relevant non major bleeding).

Others trials are also ongoing to compare LAAO using various devices: Amplatzer Amulet versus Watchman devices.

The AMPLATZER Amulet LAA Occluder trial (Amulet IDE) is a RCT that has recruited AF patients with high ischemic risk, randomized to percutaneous LAAO with the Amplatzer Amulet versus Watchman. The primary safety endpoint is a composite of procedure-related complications, all-cause death or major bleeding up to 12 months, while the primary efficacy endpoint is a composite of ischemic stroke and/or SE up to 18 months.

## 8. LAAO in Clinical Practice: Current Indications and Patients’ Selection

The latest ESC guidelines [1] on AF recommend anticoagulants as the first line treatment for prevention of stroke and SE in males with a CHA2DS2-VASc score ≥2 and in females with a score ≥3 (class of recommendation I, level of evidence A). On the other hand, anticoagulant therapy should be considered as a class IIaB indication in males with a score of 1 and in females with a score of 2. In patients eligible for OAC, DOACs represent the first-choice therapy; conversely, VKAs are used in patients with mechanical heart valves or with moderate-to-severe mitral stenosis. Percutaneous LAAO may be considered for stroke prevention in AF patients with contraindications for long-term OAC treatment (IIbB). Alternatively, in AF patients who will undergo cardiac surgery, surgical ligation or exclusion of LAA may be an option (IIbC). However, 2020 EHRA/EAPCI expert consensus [26] proposes a broader spectrum of patients in which percutaneous LAAO could be considered, after a risk-benefit assessment:

- Patients at high risk of bleeding under chronic anticoagulant therapy. This category includes: patients with HAS-BLED ≥3, patients whose bleeding risk is underestimated by the HAS-BLED (e.g., tumors, thrombocytopenia), patients with prolonged triple antithrombotic therapy. Patients with previous major bleeding from gastrointestinal tract, with a source who cannot be eliminated (e.g., diffuse intestinal angiodysplasia) [72].
- Patients with end-stage CKD or on hemodialysis treatment. DOACs are contraindicated when creatinine clearance is <15 mL/min, while warfarin could cause tissue calcifications in these patients. A meta-analysis [73] highlighted that in AF patients with end-stage CKD, warfarin could raise the risk of major bleedings. Thus, patients with end-stage CKD would be suitable candidates for LAAO. However, no data from RCTs are available for this specific population.
- Non-compliance to long-term medical therapy (dementia, patients who discontinue drug after a minor bleeding event) or patients with difficulties in managing oral therapies (e.g., visual impairment, psychiatric diseases). In this category, LAAO could be considered only after attempting to improve patient’s compliance.
- Patients in whom OACs were not able to prevent cerebral ischemic events likely related to thrombus-embolism from LAA. This group includes patients developing an ischemic stroke event despite adequate OAC therapy. A number of studies (Table 2) have evaluated the rate of ischemic stroke on OACs varying from 1.69%, 2.2%, and 1.05% using warfarin, to 1.53% with dabigatran, 1.7% with rivaroxaban and 1.27% with apixaban [54,55,56]. In this context, percutaneous LAAO could represent a potential alternative to medical therapy. However, data are insufficient to provide specific recommendations [26].
- Patients who underwent electrical isolation of LAA as part of AF ablation procedure due to an increased risk of stroke. Evidence is still scarce and there are no trials comparing DOACs with percutaneous LAAO in these patients [74].
- Combination of AF ablation and LAAO. AF patients that undergo ablation with a high risk of bleeding may benefit from combining these two procedures. Ablation requires a transeptal approach that allows percutaneous LAAO using the same access. Small cohorts have demonstrated feasibility, but there are no trials comparing a combined procedure versus a two-step procedure [75].
- LAAO for “primary prevention”. In patients with ASD and at high risk to develop AF, LAAO could be used before closing septal defects due to future technical problems related to the presence of septal devices [76]. More data are needed to better evaluate the adequacy of this indication.

## 9. Conclusions

LAAO has shown to be a safe and effective procedure for cardioembolic stroke prevention in patients with AF. Nevertheless, as it has been shown in several clinical trials, given the potential complications related to the procedure, a precise and appropriate selection of patients undergoing this procedure is essential to achieve an optimal result. Particularly, the indication for intervention must be driven mainly by the bleeding risk of the patient under an adequate anticoagulation treatment. RCTs on LAAO are still ongoing; The results are much needed to improve patients’ selection and evaluate the long-term efficacy and safety of this procedure.

## Figures and Tables

**Table 1:** Percutaneous LAAO devices currently available and approval status.

Devices	Manufacturer	Type	Size Range	Approval Status
Watchman	Boston Scientific	Endocardial	21–33	Y(CE/FDA)
ACP ^1^	Abbott Vascular	Endocardial	16–30	Y(CE)
Amulet	Abbott Vascular	Endocardial	16–34	Y(CE)
WaveCrest	Biosense Webster	Endocardial	22–32	Y(CE)
LAmbre LAAO	Lifetech Scientific	Endocardial	16–26/36	Y(CE)
Occlutech	Occlutech	Endocardial	15–39	Y(CE)
Ultraseal	Cardia	Endocardial	16–32	N
Sideris Patch	Custom Medical Devices	Endocardial	<25	N
Pfm	Pfm Medical	Endocardial	15–25	N
Lariat	SentreHEART	Epicardial	40	Y(CE/FDA)
Sierra Ligation System	Aegis Medical Innovation	Epicardial	One size	N

^1^ ACP indicates Amplatzer Cardiac Plug; CE, Conformité Européenne; FDA, Food and Drug Administration; Y, Yes; N, No.

**Table 2:** An overview of main RCTs evaluating efficacy and safety of OACs.

RCT	DOACs **	Warfarin	Median Follow-Up (Years)	Efficacy *^,†^			Hemorrhagic Stroke *		
				DOACs	DOACs	Warfarin	DOACs	DOACs	Warfarin
				High-Dose	Low-Dose		High-dose	Low-Dose	
ARISTOTLE	9.120	9.081	1.8	1.27	NA	1.60	0.24	NA	0.47
ENGAGE AF-TIMI 48	14.069	7.036	2.8	1.18	1.07	1.50	0.26	0.16	0.47
RELY	12.091	6.022	2	1.53	1.11	1.69	0.10	0.12	0.38
ROCKET-AF	7.131	7.133	1.6	1.7	NA	2.2	0.5 ***	NA	0.7 ***

* events per 100 patient-years, NA: Data not available, ** DOACs: Apixaban 5 mg × 2/die (ARISTOTLE); Edoxaban 60 mg/die (high-dose) or 30 mg/die (low-dose) (ENGAGE AF-TIMI 48); Dabigatran 150 mg/die (high-dose) and 110 mg/die (low-dose) (RELY); Rivaroxaban 20 mg/die (ROCKET-AF). ^†^ Efficacy: stroke or systemic embolism (ARISTOTLE, ENGAGE AF-TIMI 48, RELY, ROCKET-AF). *** In ROCKET-AF trial, data are available only for intracranial hemorrhages.

**Table 3:** An overview of the main RCTs evaluating efficacy and safety of LAAO.

RCT	Device	Control	Mean Follow-Up (Months)	Efficacy *^,†^		Safety *^,ɬ^		Implant Success
				Device	Control	Device	Control	
PROTECT-AF	463	244	45 ± 20	2.3	3.8	3.6	3.1	90.9%
PREVAIL	269	138	11.8 ± 5.8	6.4	6.3	2.2	NA	95.1%
PRAGUE-17	201	201	20.8 ± 10.8	10.99	13.42	NA	NA	95.5%

* events per 100 patient-years, NA: Data not available, ^†^ Efficacy endpoint: stroke, systemic embolism or cardiovascular/unexplained death (PROTECT-AF and PREVAIL); stroke/TIA, systemic embolism, cardiovascular death, bleeding, device-related complications (efficacy and safety endpoint in PRAGUE-17). ^ɬ^ Safety endpoint: Major bleeding or procedure-related complications (PROTECT-AF); all-cause death, ischemic stroke, SE or procedure-related events requesting surgery, occurred within seven days (PREVAIL).

## Data Availability

Data available in a publicly accessible repository, the data presented in this study are openly available in PubMed.
